# Supplementary material for: The AAA-ATPase Ter94 regulates wing size in Drosophila by suppressing the Hippo pathway
Source: Commun Biol. 2024 May 6;7:533. doi: 10.1038/s42003-024-06246-x (PMC11074327; doi:10.1038/s42003-024-06246-x)

Supplementary Information

The AAA-ATPase Ter94 regulates wing size in *Drosophila* by suppressing the Hippo pathway

Mingming Li et.al.

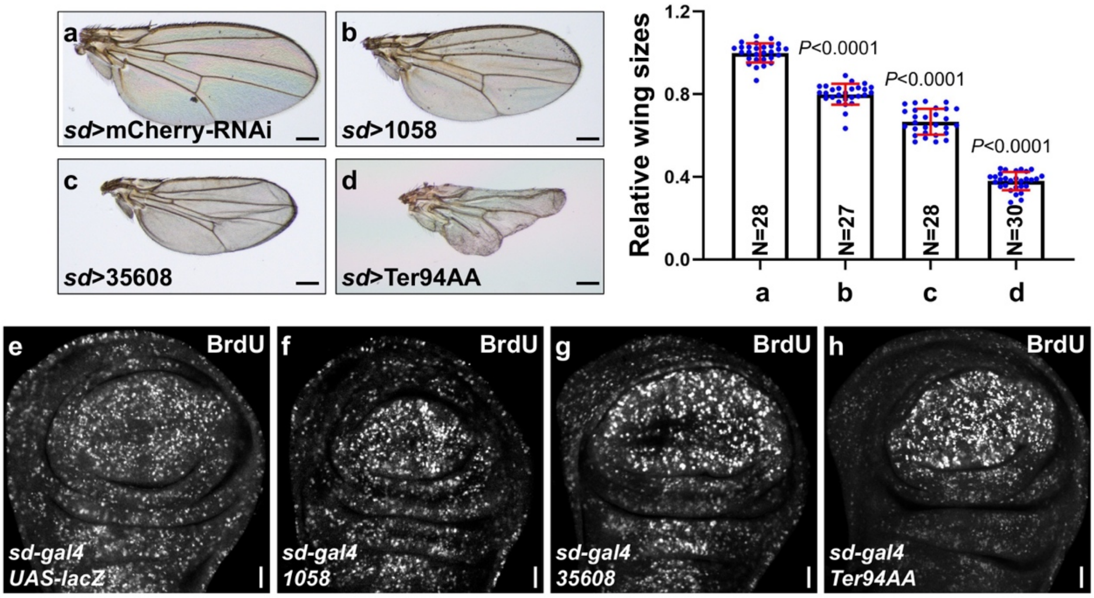

Supplementary Fig. 1 Knockdown of *ter94* reduces wing sizes. a-d Comparison of adult wings from control (**a**), *ter94* knockdown by *sd*-gal4 (**b**, **c**), and expressing Ter94AA via *sd*-gal4 (**d**). Quantification analyses of relative wing sizes were shown on right. The numbers in the bars represented the number of wings counted. **e** A control wing disc expressing UAS-lacZ via *sd*-gal4 was stained to show BrdU incorporation (white). **f-g** Wing discs with *ter94* knockdown were stained to show BrdU incorporation. **h** Overexpression of Ter94AA elevated BrdU level. Scale bars: 200 μm for all adult wings, 20 μm for all wing discs.

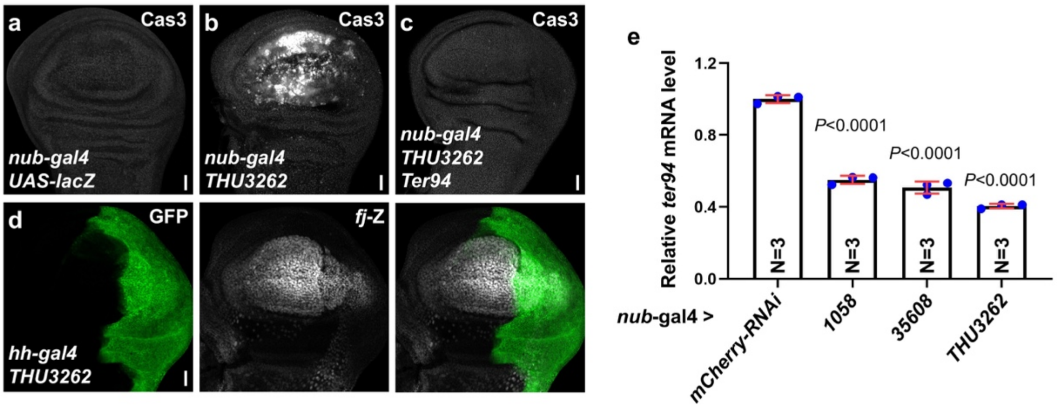

Supplementary Fig. 2 Analyses the knockdown efficiencies of the distinct RNAi lines. a-c A wing disc expressing THU3262 (*ter94*-RNAi) driven by *nub*-gal4 activated Cas3 (**b**) compared to the control disc (**a**). Co-expression of Ter94 and THU3262 rescued the increased Cas3 (**c**). **d** Knockdown of *ter94* (THU3262) decreased *fj*-lacZ level. **e** Relative mRNA levels of *ter94* from wing discs were revealed by RT-qPCR. Scale bars: 20 μm for all wing discs.

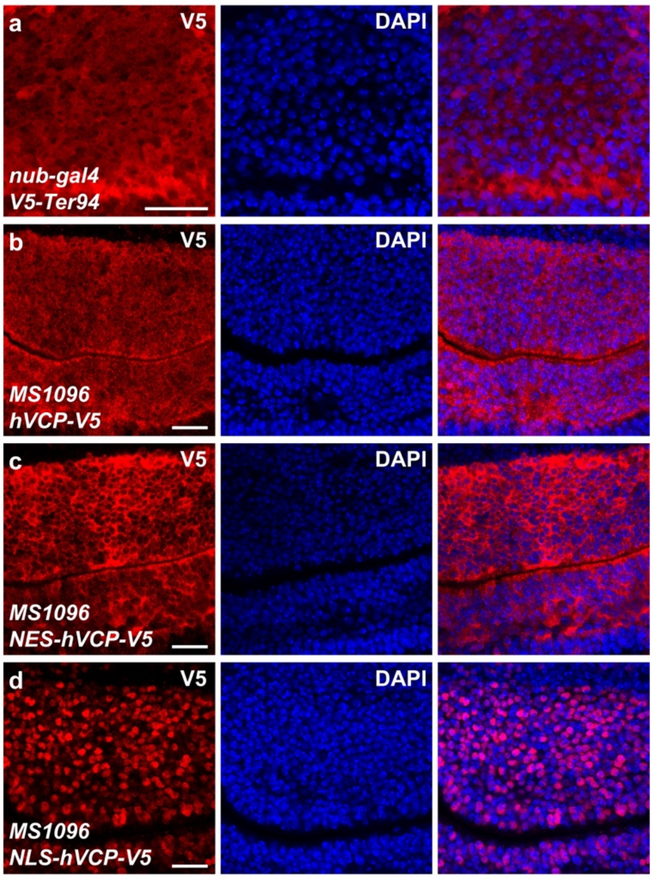

Supplementary Fig. 3 Analyses the subcellular localization of Ter94 and hVCP. a A wing disc expressing V5-Ter94 by *nub*-gal4 was stained to show V5 (red) and DAPI

(blue). DAPI staining marks the nuclei. Of note, V5-Ter94 localizes in the cytoplasm and nucleus. **b** A wing disc expressing hVCP-V5 by MS1096-gal4 was stained to show V5 (red) and DAPI (blue). hVCP resides in both the cytoplasm and nucleus. **c** NES-hVCP-V5 exclusively localized in the cytoplasm. **d** NLS-hVCP-V5 localized in the nucleus. Scale bars: 20 μm for all wing discs.

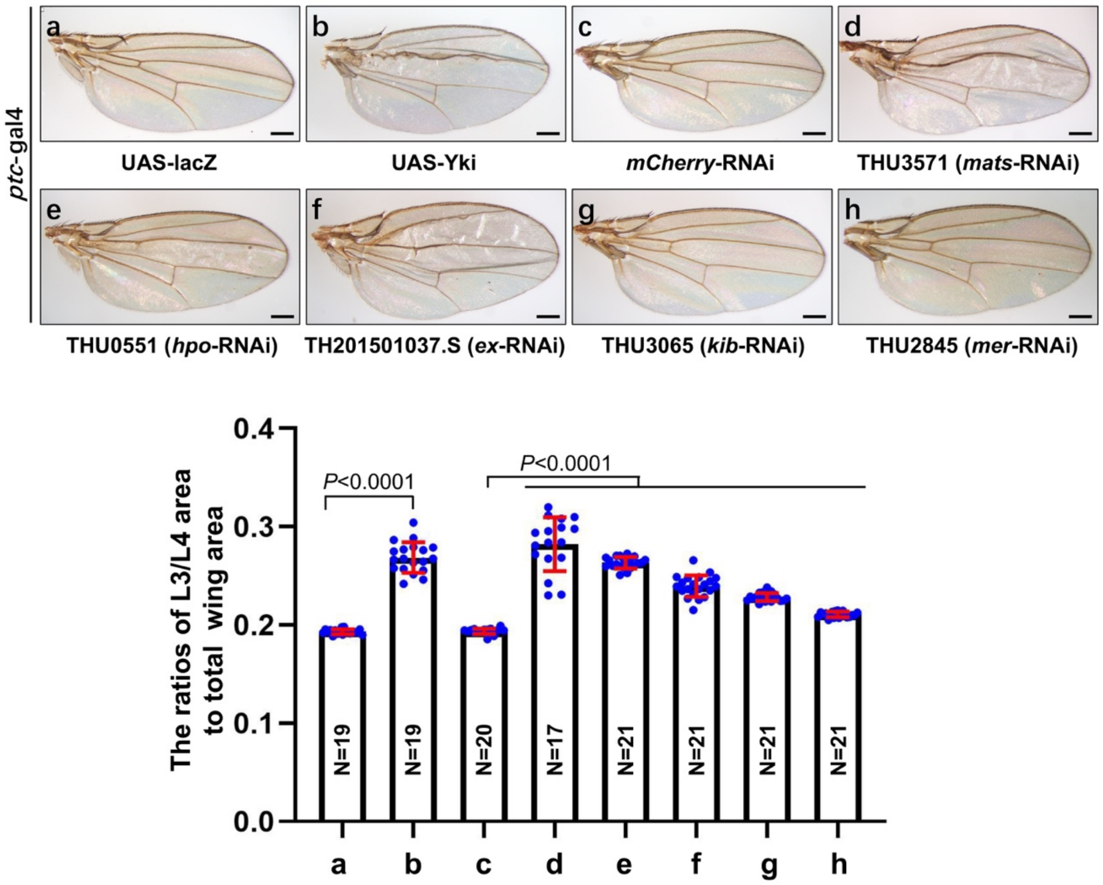

Supplementary Fig.4 Verification the efficiencies of Hippo pathway-related flies. **a**-**b** Adult wings expressing lacZ by *ptc*-gal4 (**a**), expressing Yki (**b**) were shown. Quantification analyses of relative wing sizes were shown below. Overexpression of Yki increases the size between vein L3 and vein L4. **c**-**h** Comparison of adult wings from control (**c**), *mats* knockdown (**d**), *hpo* knockdown (**e**), *ex* knockdown (**f**), *kib* knockdown (**g**), and *mer* knockdown (**h**). Quantification analyses of relative wing sizes were shown below. The numbers in the bars represented the number of wings counted. Notably, knockdown of the Hippo pathway components using *ptc*-gal4 elevated the width between vein L3 and vein L4. Scale bars: 200 μm for all adult wings.

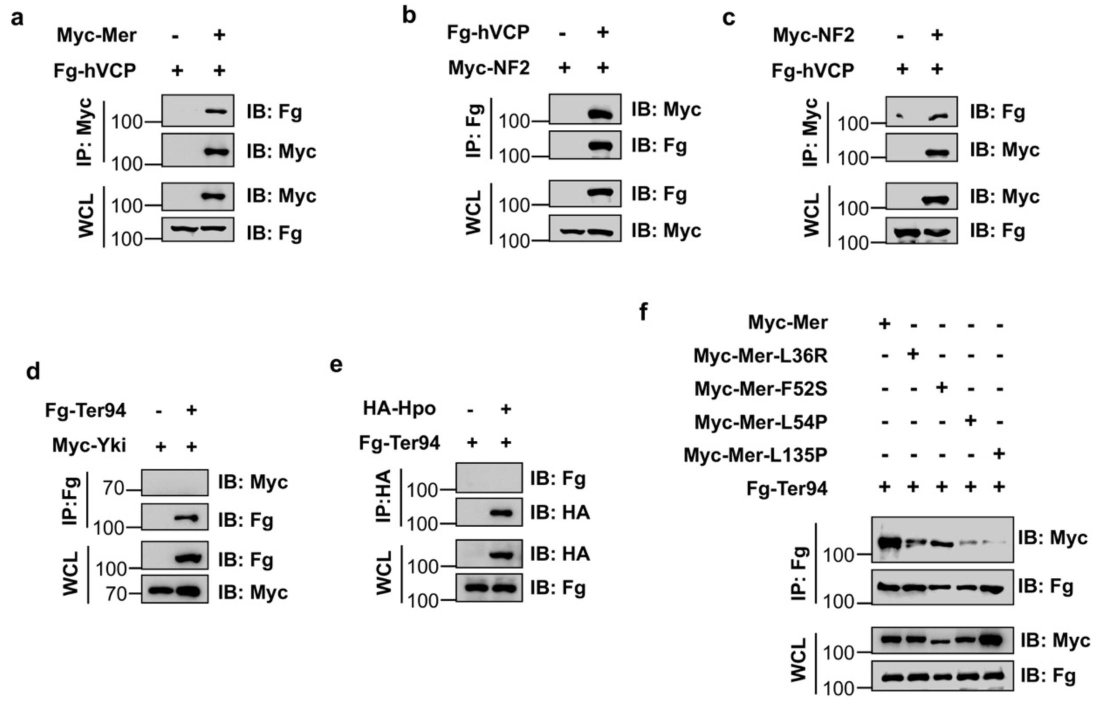

Supplementary Fig.5 The interaction of Ter94 with Mer is conservation. **a** Myc-Mer pulled down Fg-hVCP in HEK-293T cells. **b-c** Fg-hVCP interacted with Myc-NF2 in HEK-293T cells. **d-e** Ter94 cannot interacts with Yki or Hpo in HEK-293T cells. **f** The FERM domain mutations in Mer weakened the interaction with Fg-Ter94.

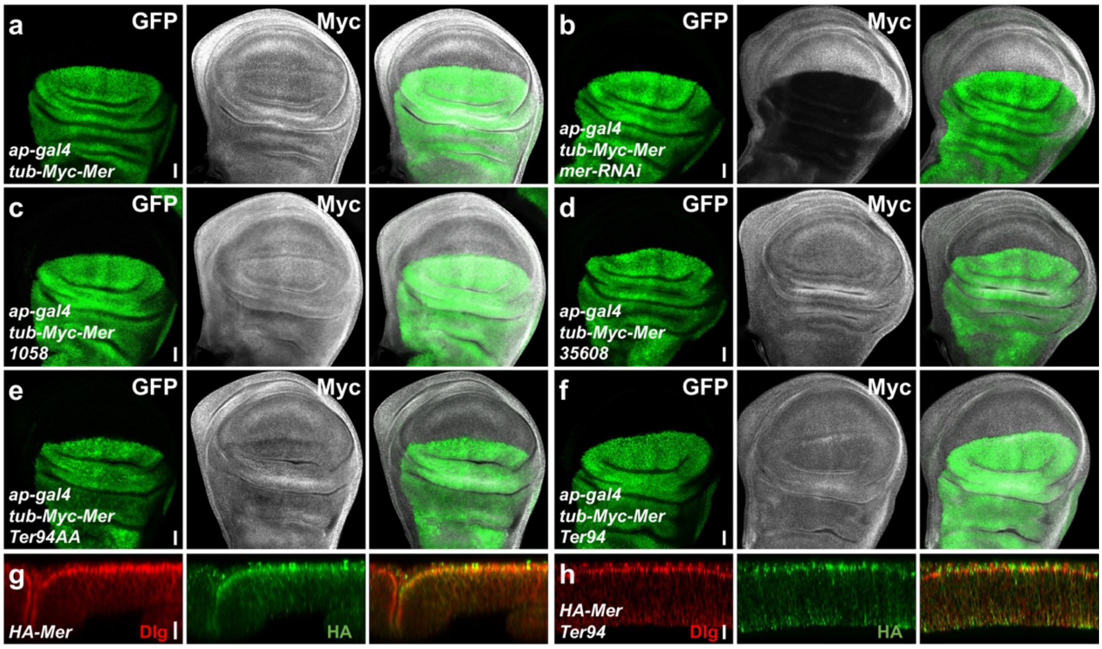

Supplementary Fig.6 Ter94 does not affect Mer protein level. a A control wing disc harboring tub-Myc-Mer was stained to show GFP (green) and Myc (white). GFP marks the expression pattern of *ap*-gal4. The Myc-Mer protein was expressed by tubulin

promoter. Of note, Myc-Mer evenly expresses in the wing disc. **b** *mer* RNAi enabled to silence the expression of *tub*-Myc-Mer. **c** Knockdown of *ter94* using 1058 failed to affect *tub*-Myc-Mer level. **d** Silence of *ter94* using 35608 did not influence *tub*-Myc-Mer protein. **e** Overexpression of Ter94AA did not alter *tub*-Myc-Mer level. **f** Ectopic expression of wild-type Ter94 failed to change *tub*-Myc-Mer level. **g-h** Cross-section (X-Z) views of Dlg (red) and HA-Mer (green) staining showed apical location (apical is up). Overexpression of Ter94 by *C765*-gal4 reduced Mer apical location (**h**). Scale bars: 20 μm for a-f images, 10 μm for g-h images.

Supplementary Fig.7

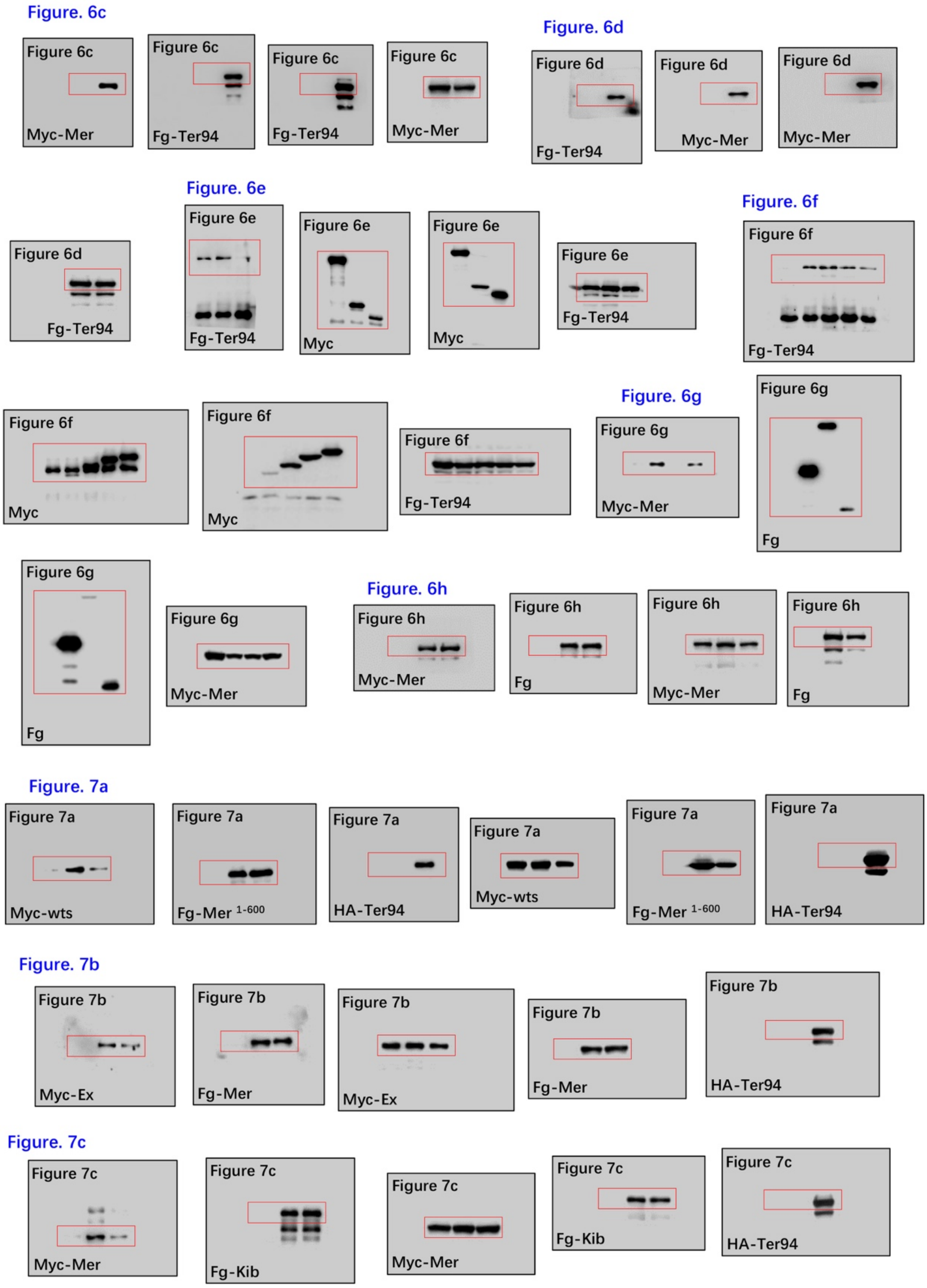

Supplementary Fig.8

Figure. 7d

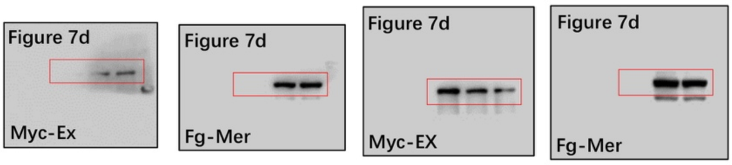

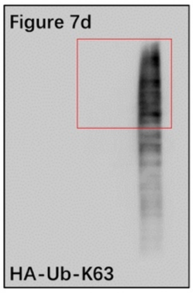

Figure. 7e

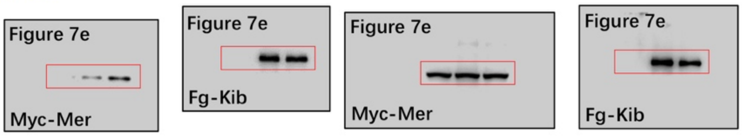

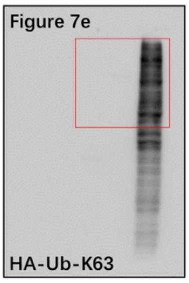

Supplementary Fig. 5a

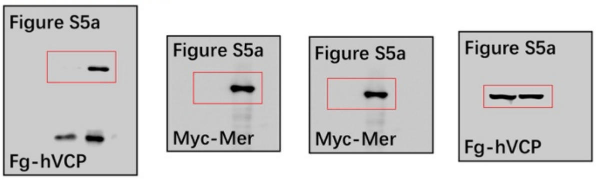

Supplementary Fig. 5b

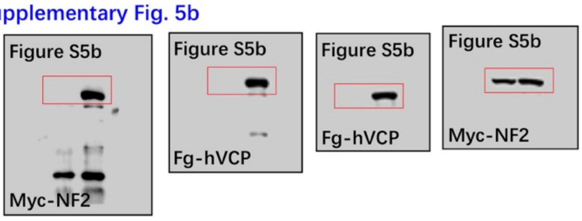

Supplementary Fig. 5c

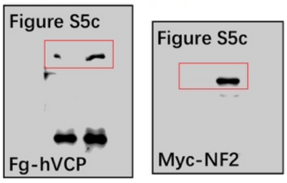

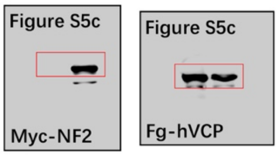

Supplementary Fig. 5d

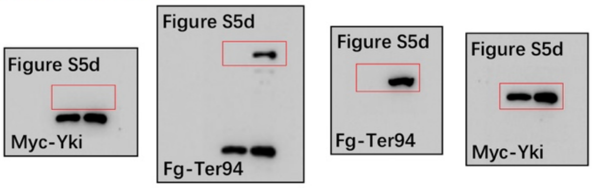

Supplementary Fig. 5e

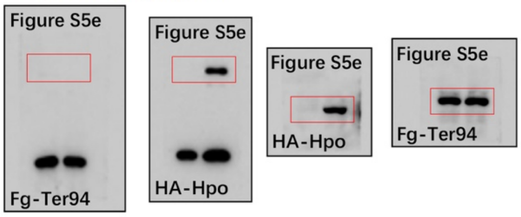

Supplementary Fig. 5f

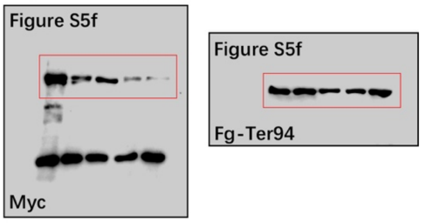

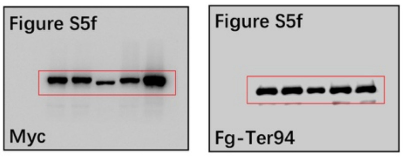

Supplement: Supplementary file 2 — Supplementary information [file 42003_2024_6246_MOESM2_ESM.pdf]
