# Supplementary material for: The AAA-ATPase Ter94 regulates wing size in Drosophila by suppressing the Hippo pathway
Source: Commun Biol. 2024 May 6;7:533. doi: 10.1038/s42003-024-06246-x (PMC11074327; doi:10.1038/s42003-024-06246-x)
Supplement: Supplementary file 3 — Description of Additional Supplementary Materials [file 42003_2024_6246_MOESM3_ESM.docx]

**Description of Additional Supplementary Files**

**File name:** Supplementary Data

**Description:** All source data underlying the graphs
